# Atrial Myxoma, a Rare Cause of Sudden Cardiac Death: A Case Report and Review of Literature

**DOI:** 10.7759/cureus.6704

**Published:** 2020-01-19

**Authors:** Mustajab Hasan, Ramy Abdelmaseih, Mohammed Faluk, Jay Chacko, Hesham Nasser

**Affiliations:** 1 Internal Medicine, Ocala Regional Medical Center/University of Central Florida College of Medicine, Ocala, USA; 2 Internal Medicine, Ocala Regional Medical Center/ University of Central Florida College of Medicine , Ocala, USA; 3 Internal Medicine, Ocala Regional Medical Center/ University of Central Florida College of Medicine, Ocala, USA; 4 Internal Medicine, University of Central Florida College of Medicine, Ocala, USA

**Keywords:** atrial myxoma, cardiology, sudden cardiac death

## Abstract

Despite the huge improvement in cardiovascular care over the past several decades and the decline in cardiovascular deaths, sudden cardiac death (SCD) continues to present a nationwide health problem accounting to more than half of all deaths from cardiovascular disease. Majority of these cases are related to coronary artery disease and arrhythmias, however, a very small number of these cases are secondary to cardiac neoplasms. These neoplasms commonly present with conduction abnormalities or symptoms secondary to valvular disease such as dyspnea, orthopnea, cough and/or edema. This is a rare case of a 63-year-old gentleman who suffered sudden cardiac death secondary to a cardiac myxoma.

## Introduction

Sudden cardiac death (SCD) is defined as the unexpected death that occurs within one hour from the beginning of symptoms when death is witnessed, or within 24 hours of being seen alive and well when it is unwitnessed, without having any other non-cardiac causes that may lead to sudden death such as: massive pulmonary embolism, intracranial hemorrhage, end stage diseases or advanced malignancy that is not in remission [1-3].

SCD refers to the sudden cessation of the cardiac mechanical activity with global circulatory and hemodynamic collapse leading to direct cellular damage, edema formation and subsequently death. Conditions implicated in the pathogenesis of SCD includes: ischemic heart diseases (coronary artery disease with myocardial infarction (MI) or angina, coronary artery embolism or spasm, vasculitis), non-ischemic heart diseases (cardiomyopathies, valvular and congenital heart diseases, acute pericardial tamponade, acute myocardial rupture, aortic dissection) and nonstructural heart diseases (primary electrical disease, Brugada syndrome, Long QT syndrome, complete heart block, familial sudden cardiac death, chest wall trauma).

Although the majority of these SCD mortalities are related to atherosclerotic coronary artery disease and arrhythmias, it has been found that most cardiac arrest survivors do not have evidence of an acute MI but previous infarcts, however, a very small percentage of these mortalities (approximately 0.001-0.3%) is linked to primary cardiac neoplasms [4-5].

Primary cardiac tumors are classified into benign tumors (myxomas, papillary fibroelastomas, rhabdomyomas, fibromas, hemangiomas, teratomas, lipomas, paragangliomas and pericardial cysts) and malignant tumors including sarcomas, pericardial mesotheliomas and primary lymphomas.

Primary cardiac tumors are exceptionally uncommon, based upon data of 22 large autopsy series, the frequency of primary cardiac tumors is approximately 0.02%, corresponding to 200 tumors in one million autopsies [6]. They may be symptomatic mimicking other cardiac conditions but most of the times are found incidentally during evaluation for an unrelated physical finding. Yet, they can be potentially lethal causing hemodynamic compromise or life threatening dysrhythmias and SCD.

## Case presentation

**Figure 1:**
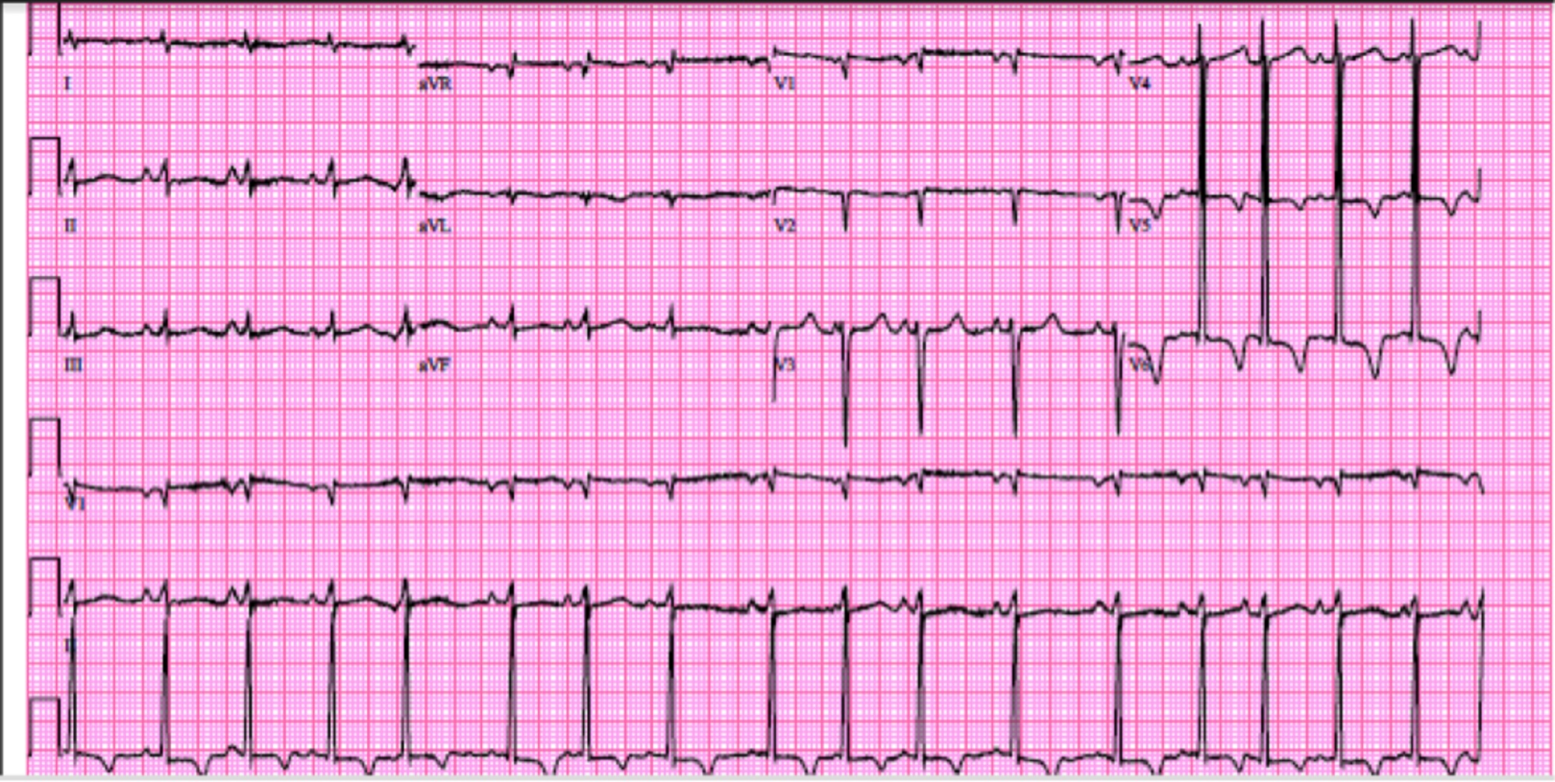
Electrocardiogram on admission.

**Figure 2:**
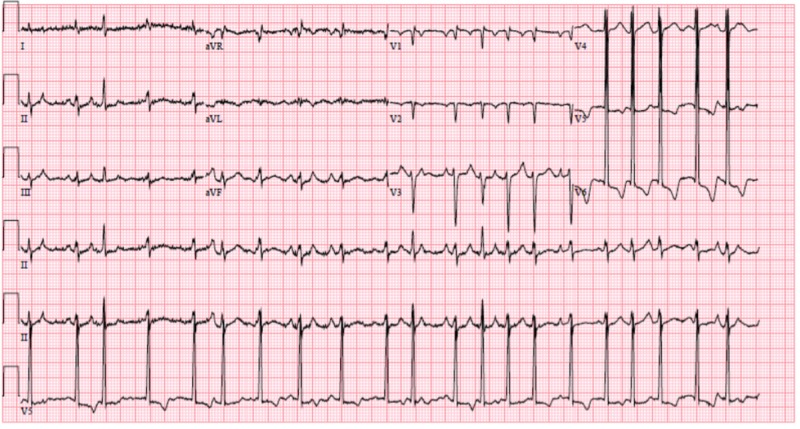
Electrocardiogram on worsening of respiratory status showing atrial fibrillation with rapid ventricular response in addition to a prolonged QTc.

A 63-year-old Caucasian gentleman who was an active smoker with a 40-pack year history and otherwise unremarkable medical history presented to the emergency department with complaints of progressively worsening shortness of breath and intermittent palpitations, which began five days before admission. Upon further questioning, the patient reported that shortness of breath was positional, aggravated by laying flat and relieved by sitting upright and leaning forward. Additionally, the patient reported nonproductive cough and generalized weakness but otherwise denied fevers, chills, nausea, vomiting, chest pain, dizziness, falls, headaches or focal deficits. Review of systems was otherwise negative. Upon arrival to the emergency department, initial electrocardiogram showed sinus tachycardia and left ventricular hypertrophy (see Figure 1), shortly thereafter the patient developed worsening hypoxic respiratory failure and palpitations. Repeat electrocardiogram showed atrial fibrillation with rapid ventricular response (see Figure 2).

**Figure 3:**
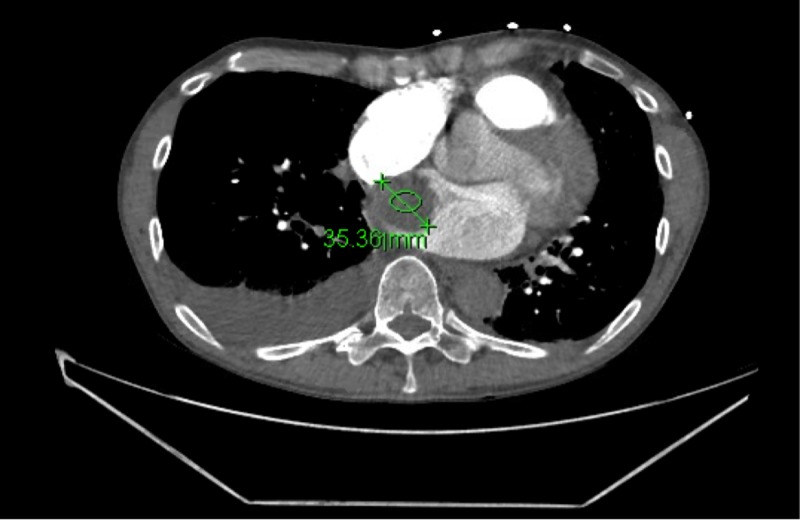
CT scan to rule out pulmonary embolism showing a 35.36 mm right atrial cardiac tumor.

Due to concern for pulmonary embolism in light of elevated D-Dimer, CT chest with contrast was done which was negative for PE, however did show bilateral pleural effusions and a 35-mm cardiac tumor with imaging characteristics of a left atrial cardiac myxoma (see Figure 3). Upon admission after extensive discussion, the patient adamantly refused any aggressive intervention, shortly after which he developed hypoxic respiratory failure. Blood pressure was 80/60 and immeasurable thereafter and the patient appeared pale due to lack of perfusion due to myxoma obstruction of mitral valve. Echocardiogram was unsuccessful due to patient refusal and shortly thereafter the patient expired. This rapid deterioration highlights the importance of diagnosing cardiac tumors promptly and being cognizant of acute complications of such presentations.

Pertinent Labs on admission were as follows (Table 1):

**Table 1:** Diagnostic labs.

Test	Value
White blood cells	15.8 x 10^3 ^/mm^3^
Lactic acid	4.75 mmol/L
B-type natriuretic peptide	34800 pg/mL
D-Dimer	584 ng/mL DDU
Liver function tests	Aspartate aminotransferase: 239
	Alanine aminotransferase: 147
	Total bilirubin 2.0
	Direct bilirubin: 0.0
Arterial blood gas	pH: 7.45
	pCO_2_: 27
	pO_2_: 60
	HCO_3_: 18.6
	O_2 _Saturation: 92%
	FiO_2_: 50%

## Discussion

Background

Myxomas are the most common primary cardiac tumors. The World Health Organization (WHO) defines a cardiac myxoma as a neoplasm composed of stellate to plump, cytologically bland mesenchymal cells set in a myxoid stroma. Myxomas resemble 50% of all cardiac tumors in adults, but about only 15% of such tumors in children [7]. Most cardiac myxomas (about 75%) arise from the left atrium and less commonly the right atrium (18%), right ventricle (3%) and very rarely from the left ventricle (3%) and only very small fraction (<1%) involves the valves. Because of the nonspecific symptoms, early diagnosis of cardiac myxomas can always be challenging.

Epidemiology

Myxomas represent about 0.25% of all cardiac diseases, 50% of all cardiac tumors and about 50-85% of all benign ones. They have been described prenatally and in individuals as old as 97 years old. They occur mainly in middle-aged population with average age 50-60 years, affecting women more frequently than men. The female to male sex ratio in left atrial cardiac myxomas was found to be 2:1 while in right atrial cardiac myxomas it was 0.75:1 [8,9].

Cardiac myxomas are generally classified into familial and sporadic types. The familial type accounts for only 5% of all cases and affects predominantly young males with mean age of 25 years. It is autosomal dominant and tumors are usually multiple and may arise in other cardiac chambers. This type is associated with Carney’s complex (a rare genetic disorder with mutation in the perinatal myosin heavy chain causing multiple cardiac and extra cardiac neoplasia in endocrine system and skin along with cutaneous pigmentation). On the other hand, the sporadic type is more predominant and accounts for 95% of the cases.

Etiology

Most cases of atrial myxomas are sporadic and the exact origin remains inadequately understood. Familial atrial myxomas are of autosomal dominant inheritance. In Carney syndrome (linked to familial type) abnormalities in short arm of chromosome 2 (Carney), chromosome 12 (Ki-ras oncogene) and protein kinase A regulatory subunit 1 alpha (PRKAR1A- the causative gene of Carney complex) frame shift mutations are implicated in the etiology.

Historically, there were numerous theories regarding the etiology and development of myxomas that were rejected afterwards. One of them believed that myxomas arise from cardiac mural thrombi, but later on it was progressively rejected based on the tremendous differences between myxomas and mural thrombi as thrombi tends to occur in many locations within the heart on top of an underlying disease. On the contrary, myxomas arise with astonishing consistency mainly in an area adjacent to the fossa ovalis without any underlying pathology. Moreover, myxomas do not organize into fibrous tissue or show stratification and behave differently from mural thrombi in tissue culture studies, all of which are the criteria that support the neoplastic nature of myxomas.

Currently, it is believed that cardiac myxoma originates from multipotent mesenchymal stem cells as its cells exhibit the phenotypic markers of embryonic endothelial-to-mesenchymal transformation and the markers of primitive cardiac mesenchymal differentiation. Furthermore, this hypothesis is supported because of the similarities between the endothelial lining cells, the cushion tissue cells and the cardiac myxoma’s lepidic cells.

Finally, based on immuno-histochemical data, pattern of neuroendocrine markers and distribution of VWF/FVIII, CD34 and smooth muscle actin, and the presence of CALB2 (calretinin/calbindin 2 - a protein normally detected in the cells of the central and peripheral neural tissue) in all examined sporadic cardiac myxoma cases, some authors support the nervous origin of myxomas from neuroendocrine tissue [10,11].

Anatomy - Pathophysiology

Cardiac myxomas arise mainly from inter-atrial septum near fossa ovalis (75% left atrium, 18% right atrium), although myxomas can also originate from the posterior atrial wall, the anterior atrial wall, or the atrial appendage and less commonly from the ventricles, bi-atrial or multifocal.

On gross anatomy, cardiac myxomas are polypoid, round, or oval that are often pedunculated and soft gelatinous in consistency with a smooth, villous, or friable surface. They are usually white, yellow or brown and weigh anywhere between 15 g and 180 g and their average size is between 2 cm and 6 cm but can reach 15 cm. On cut section, myxomas have a variegated appearance, with areas of pearly white and yellow tissue punctuated by patchy dark-red hemorrhage, necrosis, or calcification. Smooth myxomas are often larger and present with obstructive symptoms, while villous and friable myxomas are responsible for embolic phenomenon.

Histologically, myxoma cells are stellated, fusiform and polygonal, occasionally multinucleate cells have an eosinophilic cytoplasm and are surrounded by a myxoid mucopolysaccharide stroma. Degenerative changes such as fibrosis, hemorrhages, calcification, cystic formation and gland formation (lithomyxoma) can occur. Myxomas have been shown to produce several growth factors, cytokines including vascular endothelial growth factor, which is responsible for induced angiogenesis and tumor growth.

On immunohistochemical staining, some cardiac myxoma cells are positive for CD31, CD34, and FVIIIAg. They can also stain positive for vimentin, desmin, smooth muscle myosin, CD56, S-100 protein, calretinin, α1 antitrypsin, and α1 antichymotrypsin [12].

Prognosis and complications

Although atrial myxomas are considered histologically benign tumors, yet they can be potentially lethal causing sudden cardiac death and if not, they can be very life threatening owing the potential of systemic and cerebral embolization. Following are among the factors associated with increased risk of embolism: the presence of atrial fibrillation, large tumor with irregular surface and an increase in left atrial diameter.

Sudden cardiac death is the most feared complication during the setting of cardiac myxomas. It may occur in 15% of the patients, caused by massive systemic, cerebral or coronary embolization or by sudden obstruction of blood flow at the mitral or tricuspid valves.

Other complications of atrial myxomas include: constitutional symptoms (fever, malaise and weight loss), congestive heart failure, pulmonary edema, cardiac arrhythmias, infections, myocardial infarction and rarely tumor rupture.

Based on several studies, the 10-year survival after cardiac myxoma removal was 96.8 ± 1.8% with excellent long-term prognosis. Early mortality rate was 2% and late mortality rate was 6.1%. Freedom from reoperation was 98.4 ± 1.3% at five years and 96.8 ± 1.8% at 10 years [13].

The recurrence rate is about 1-3% in sporadic myxomas but as high as 10-20% in the familial type due to abnormal DNA ploidy patterns [14].

After surgical resection, regular follow-up with echocardiography is highly recommended.

Clinical presentation

Symptoms of cardiac myxomas range in a wide spectrum from being nonspecific and constitutional to mimicking other cardiac conditions and eventually sudden cardiac death, depending on myxomas base, shape, size and mobility. About 15% of myxomas are asymptomatic, constitute of small tumors and discovered as an incidental finding during a routine examination. Cardiac conditions of mitral stenosis, tricuspid stenosis, endocarditis, mitral regurgitation can mimic atrial myxomas, hence a high index of suspicion is needed [15].

Signs and symptoms of atrial myxomas include:

- Left heart failure: dyspnea on exertion (75%), paroxysmal nocturnal dyspnea, orthopnea or pulmonary edema.

- Right heart failure: venous hyper pressure, lower limb edema, and hepatomegaly.

These symptoms are most of the time progressive with the increase of tumor size, but may have a positional character based on the movement of the cardiac tumor.

Atrial myxomas can also compromise systemic or pulmonary venous drainage, progressively or intermittently obstruct the passage of blood from atria to ventricles mimicking mitral or tricuspid stenosis and can cause dizziness, syncope, malaise or sudden cardiac death. This intra-cardiac obstruction is found in approximately 50% of cases. On the other hand, myxomas can hinder valves closure or even damage valves leaflet causing atrioventricular regurgitation. Several valvular destruction mechanisms have been reported including mechanical by the tumor itself, chemical or infectious.

Embolic Phenomena

This is one of the serious complications of atrial myxomas that is related to the process of migration of the tumor, its fragments or thrombi and vegetations adherent to the tumor surface. Embolic phenomena were found in patients with pedunculated cardiac myxomas, myxomas involving mitral valve tissue as well as myxomas prolapse through mitral valve. About 45% of left atrial myxomas get complicated with systemic emboli, most commonly to central nervous system (more than 50% of cases) resulting in transient ischemic attacks, strokes and seizures. Other sites of emboli include retinal, renal, mesenteric, coronaries, aortic and lower limbs. Right atrial myxomas can cause massive pulmonary embolism or pulmonary arterial obstruction with subsequent pulmonary hypertension [16].

Constitutional symptoms include fever, weight loss, Raynaud's phenomenon, arthralgia and myalgia, chronic anemia, thrombocytopenia or inflammatory syndromes (elevated C-reactive protein [CRP] and erythrocyte sedimentation rate [ESR], leukocytosis and hypergammaglobulinemia).

All these symptoms reflect the immune reaction against the tumor. They completely resolve after complete surgical resection of the tumor tissue.

Infection

Myxomas can get infected and become more friable increasing the risk of embolic phenomena. Most frequently isolated bacteria is Streptococcus; other organisms include Enterococcus feacalis, Staphylococcus lugdunensis, Gemella morbillorum, Porphyromonas asaccharolytica, Candida albicans and Histoplasma capsulatum [17].

Physical examinations and findings may include:

- Most commonly, a tumor plop, produced by the impact of the tumor against the endocardial wall causing an early diastolic sound.

- A rumbling diastolic atrial murmur if the tumor is obstructing the mitral or the tricuspid valves.

- A systolic murmur at the apex (mitral regurgitation) or a holosystolic murmur to the left of the sternum (tricuspid regurgitation) if the valves are damaged by tumor.

- An S 3 or S 4 may be audible.

- A loud S 1 due to the delay in mitral valve closure due to the prolapse of the tumor into the mitral valve orifice (simulating mitral stenosis).

- P 2 may be delayed with normal or increased intensity, depending on the presence of pulmonary hypertension.

- General examination may reveal increased jugular venous distention, fever, cyanosis, digital clubbing, rash, or petechiae.

- Patients with Carney syndrome may have cardiac myxomas as well as myxomas in skin, breast, thyroid gland, spotty pigmentations, endocrine hyperactivity such as Cushing syndrome and multiple cerebral fusiform aneurysms.

Other rare presentations of cardiac myxomas include:

- NAME syndrome: Nevi, Atrial myxoma, Myxoid neurofibroma, and Ephelides

- LAMB syndrome: Lentigines, Atrial myxoma, and Blue nevi.

Differential diagnoses

Atrial cardiac myxomas mimic a variety of cardiac conditions including mitral regurgitation, mitral stenosis, tricuspid regurgitation, tricuspid stenosis, pulmonary embolism and idiopathic pulmonary hypertension. Physicians should recognize any associated valvular damage and differentiate myxomas from atrial thrombi.

Other conditions to consider while suspecting atrial myxomas are carcinoid heart disease, collagen vascular disease, left atrial thrombus and cerebrovascular accident.

Patients with familial myxoma syndromes should be offered screening with transthoracic echocardiography as well as their family members as the risk of recurrence is increased in this specific type of myxomas.

Diagnostic approach

Workup of cardiac myxomas includes specific and nonspecific studies. Nonspecific studies are not diagnostic for atrial myxomas but often found in most of the cases: elevated ESR, CRP and serum gamma globulin levels, leukocytosis, anemia and serum interleukin 6 levels.

Electrocardiogram (ECG) diagnostic significance is very limited and may show atrial fibrillation, flutter, abnormal P waves or bundle branch block. Similarly, chest radiograph does not have any extra value in the diagnosis of cardiac myxomas other than showing cardiomegaly secondary to atrial enlargement.

Currently, echocardiography remains the cornerstone of diagnosing atrial myxomas. Although trans-esophageal echocardiography is more sensitive (up to 100% due to proximity of esophagus to heart, lack of intervening structures, and high-frequency imaging transducers) and specific for such cases, trans-thoracic echocardiography is usually adequate and most commonly used for the diagnosis giving the tumor location, shape, size, attachment and mobility. It also can determine the hemodynamic consequences of atrial myxomas regarding valvular stenosis or regurgitations and typically provide all necessary information prior to surgical removal.

Other diagnostic imaging includes T1-weighted cardiac magnetic resonance imaging (MRI) which provides useful information regarding the size, shape, surface and mobility of a tumor. It also provides data about tumor’s point of attachment with a postsurgical correlation of 83%.

Computed tomography (CT) scanning can also provide data about the tumor and its attachment point with postsurgical correlation of 30% (inferior to MRI). Additionally, it evaluates coronary arteries disease, cardiac structure and can add a high value in differentiating myxomas from intracardiac thrombi. Coronary CT angiography is another modality used for determining the tumor’s blood supply, which is vital information for successful excision of such tumors.

Another used imaging study is the ultra-fast CT, positron emission tomography (PET) scanning, however, it is not typically indicated in the evaluation of myxomas and only used if the diagnosis of the tumor remains unclear after an echocardiography evaluation [17].

Additionally, in suspected familial cases of Carney complex, PRKAR1 molecular genetic testing should be performed to confirm the diagnosis. Positive patients should be evaluated of cardiac myxomas, endocrine abnormalities and skin neoplasia and pigmentations.

Biopsy is not needed because imaging studies can distinguish benign tumors from malignant ones, and biopsy may spread cancerous cells in patients with a malignant primary tumor.

Treatment

Once a provisional diagnosis of atrial myxoma has been made based on imaging studies, simple tumor resection by median sternotomy and cardiopulmonary bypass is the gold standard treatment of choice because of the increased risk of systemic embolization, cardiovascular complications and sudden cardiac death.

Surgical approach for atrial myxoma resection should be safe and efficient, providing adequate exposure for complete tumor resection with minimalist tumor manipulation and allowing for inspection of all 4-heart chambers to exclude the presence of further tumors and minimize the recurrence. Cardiopulmonary bypass using the heart-lung machine is to avoid dislodging any tumor material and causing systemic embolization.

Minithoracotomy with robotic surgery resulted in shorter hospital stay, with no significant difference in the quality of life between both strategies. Endoscopic cardiac tumor resection using the port access approach in 27 cases reported that follow-up did not show any recurrent tumor. Endoscopic robotic resection of a left atrial myxoma is also reported and demonstrated good outcome [18,19].

Last resort treatment options for recurrent tumors especially in young adults include the implantation of a total artificial heart and heart transplantation provided that there are no metastases. The principal complications following successful implantation are thrombosis, infections and hemorrhage.

Postsurgical recovery is often rapid with operative mortality of 0.5%, early postoperative mortality of 2%. However, postoperative atrial arrhythmias and atrioventricular conduction abnormalities are seen in 23-33% of cases and postsurgical neurologic complications were seen in 3% of cases, and exploration for bleeding was required in 5% of cases. Long-term prognosis is excellent. Risk of recurrence was calculated to be 2-5% post-surgically, most commonly attributed to family predisposition, tumors of unrecognized multicentric origin, incomplete resection of tumor, intraoperative dissemination of tumor cells, growth from second focus and the de novo proliferation of the pre-tumor or reserve cells present in the endocardium. Biannual echocardiograms are useful for early detection of recurrent tumors [20].

## Conclusions

Left atrial myxomas are the most common type of primary cardiac tumors. They often cause a wide range of clinical signs and symptoms that mimic other cardiac conditions. Symptoms can be classified in constitutional, obstructive or embolic. The most feared complication is sudden cardiac death. Hence, a high index of suspicion, early diagnosis with appropriate imaging studies and a prompt surgical resection are very important in saving these patients’ lives.
